# A validated analytical method to measure metals dissolved in deep eutectic solvents†

**DOI:** 10.1039/d3ra02372a

**Published:** 2023-05-16

**Authors:** Halimeh Askari Sabzkoohi, Vicky Dodier, Georgios Kolliopoulos

**Affiliations:** a Department of Mining, Metallurgical, and Materials Engineering, Université Laval 1065 Av. de la Médecine Québec G1V 0A6 Canada Georgios.Kolliopoulos@gmn.ulaval.ca

## Abstract

This work presents the first validated method to analyze metals dissolved in deep eutectic solvents (DES) on a microwave plasma atomic emission spectrometer (MP-AES), which is key to the success of the upcoming field of solvometallurgical processing. The method was developed and validated for eleven metals: alkali metals: lithium (Li); alkaline earth metals: magnesium (Mg); transition metals: iron (Fe), cobalt (Co), nickel (Ni), copper (Cu), zinc (Zn), palladium (Pd); and post-transition metals: aluminum (Al), tin (Sn), and lead (Pb) in choline chloride based DES. The proposed method was validated with respect to linearity, limit of detection (LOD), limit of quantification (LOQ), accuracy, precision, and selectivity. Our method's selectivity was evaluated for three DES matrices: (1) choline chloride: ethylene glycol, (2) choline chloride: levulinic acid, and (3) choline chloride: ethylene glycol in the presence of iodine, which is an oxidant often used in solvometallurgy. In all three matrices, the linearity range was plotted with at least 5 levels of standard solutions. All the parameters satisfied the acceptability criteria suggested by international organizations, such as the International Council for Harmonization, AOAC International, and the International Union of Pure and Applied Chemistry. Specifically, the calculated LOD and LOQ are comparable with aqueous matrices on MP-AES and with other analytical methods. The metal with the lowest reported LOD (0.003 ppm) and LOQ (0.008 ppm) was Cu, while the highest LOD and LOQ were obtained for Mg at 0.07 and 0.22 ppm, respectively. The recovery and precision for the three DES matrices were acceptable, *i.e.*, between 95.67–108.40% and less than 10%, respectively. Finally, to compare the proposed method with the standard analytical method used to measure metals dissolved in aqueous solutions, we used 2 ppm standard solutions in DES and found that the accuracy was unacceptable without using the proposed method. Therefore, it is evident that our method will be pivotal in the field of solvometallurgy, as it will allow accurate and precise detection and quantification of metals dissolved in DES and eliminate quantification errors, which were estimated in excess of 140% without using the method developed and proper DES matrix-matched calibrations.

## Abstract 2

This work presents the first validated method to analyze metals dissolved in deep eutectic solvents (DES) on a microwave plasma atomic emission spectrometer (MP-AES), which is key to the success of the upcoming field of solvometallurgy.

## Graphical abstract

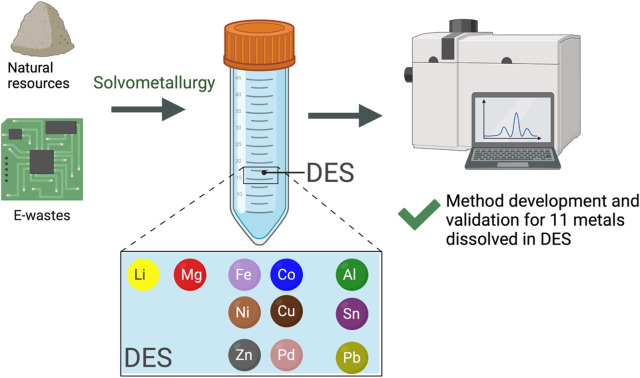

## Introduction

Metal extraction and recovery are performed by pyrometallurgical and hydrometallurgical processes for the treatment of high-grade and low-grade metal resources, respectively.^1^ These conventional extractive metallurgy techniques are generally energy, water, and acid/base chemical intensive, leading to the generation of large quantities of waste off-gases, including greenhouse gases, and aqueous effluents, which need to be treated for reuse in process circuits or disposal in tailings ponds.^1,2^ Therefore, cleaner alternatives for metal production ought to be developed to minimize the use of water and energy as well as the generation of waste by-products. Solvometallurgy has been proposed as such a potential alternative as it replaces water with non-aqueous deep eutectic solvents (DES), thus eliminating or minimizing the use of water in process circuits.^1,3,4^ The number of DES-related publications has increased dramatically in recent years.^5–9^ However, an accurate and precise analytical method to determine the concentration of metals dissolved in DES is still lacking. Such a method could facilitate the transition to a solvometallurgical processing future, as it would ensure the accurate and precise measurement of dissolved metals while avoiding incorrect interpretations and unwarranted conclusions based on inaccurate experimental data.^10^

The most frequent methods used for measuring metals in aqueous solutions are based on spectroscopy,^11,12^ which include flame and graphite furnace atomic absorption spectroscopy (F-AAS and GF-AAS), inductively coupled plasma atomic emission spectroscopy (ICP-AES) and mass spectroscopy (ICP-MS), and microwave plasma atomic emission spectroscopy (MP-AES).^13^ F-AAS and GF-AAS are widely used due to their simplicity, low cost, and effectiveness and have been used to determine metal concentration in some hydrometallurgical processes.^14,15^ However, they can only analyze one element at a time, while also suffering from low sensitivity,^16^ which are considerable drawbacks. ICP-AES, also referred to as inductively coupled plasma optical emission spectroscopy (ICP-OES), is commonly used for metal analyses.^17,18^ Its popularity can be attributed to its high stability, multi-elemental analysis, low noise, and low background noise.^12^ However, ICP-based analytical methods are expensive and complicated.^12^ A cost-effective and relatively simpler alternative to ICP is MP-AES, which may be used for determining the concentration of dissolved metals.^10^

MP-AES has been applied successfully for the quantification of metals in various matrices.^19–21^ However, to-date, there is no validated analytical method to determine the concentration of metals dissolved in DES, which is primarily done using ICP-AES.^22–29^ DES are a cheap alternative to ionic liquids that typically consist of a mixture of choline chloride and a hydrogen bond donor chemical.^1^ The lack of a validated analytical method for metals dissolved in DES means that the matrix effects for DES on the measurement of metals by spectroscopy have yet to be studied. Therefore, a validated method, especially with respect to selectivity (*i.e.*, effect of matrix), to measure metals in different DES is needed.

**Fig. 1:**
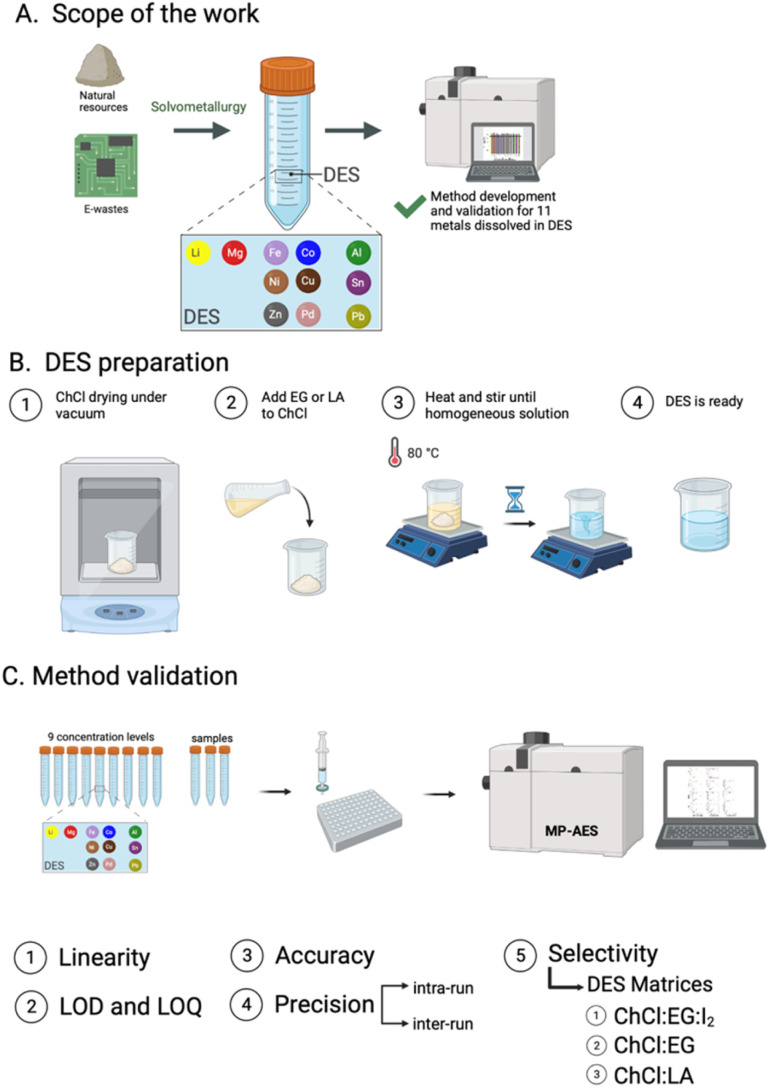
(A) Scope of this work and potential applications of the proposed method. (B) DES preparation. (C) Validation parameters for dissolved metal analysis in DES.

The objective of the current study was to develop and validate an analytical method to measure metals dissolved in DES using MP-AES. Three choline chloride (ChCl)-based DES matrices and eleven metals, including alkali metals: lithium (Li); alkaline earth metals: magnesium (Mg); transition metals: iron (Fe), cobalt (Co), nickel (Ni), copper (Cu), zinc (Zn), palladium (Pd); and post-transition metals: aluminum (Al), tin (Sn), and lead (Pb), were studied. Ethaline, which is one of the most common DES consisting of ChCl and ethylene glycol (EG), as well as ChCl:levulinic acid (LA) were tested. The chosen DES were selected due to their relatively low viscosity *i.e.*, 36 cP for ChCl:EG at 25 °C ^9^ and 320 cP for ChCl:LA at 20 °C (H_2_O = 0.5 wt%)^27^ compared to other commonly used DES, such as ChCl:urea with a viscosity of 632 cP at 25 °C.^9^ The lower viscosity of the DES could facilitate their use in solvometallurgical process circuits. Further, the performance of our method was assessed with the addition of iodine (I_2_), which is a commonly used oxidant in solvometallurgy,^12^ to the ethaline DES matrix. The developed method was validated with respect to linearity, limit of detection (LOD), limit of quantification (LOQ), accuracy, precision, and selectivity. The proposed method is expected to be a universal tool for measuring dissolved metals in DES and thus help the transition to solvometallurgical processes (Fig. 1A).

## Experimental

### Chemicals

Choline chloride (ChCl, (CH_3_)_3_N(Cl)CH_2_CH_2_OH, 99%), ethylene glycol (EG, C_2_H_6_O_2_, >99%), levulinic acid (LA, CH_3_COCH_2_CH_2_COOH, 99%), iodine (I_2_, >99.8%), and a single element calibration solution for lithium (Li, 998 ± 4 μg mL^−1^) were purchased from Sigma-Aldrich. The single element calibration solutions for magnesium (Mg, 999 ± 4 μg mL^−1^), iron (Fe, 1000 ± 4 μg mL^−1^), cobalt (Co, 1002 ± 4 μg mL^−1^), nickel (Ni, 1003 ± 4 μg mL^−1^), copper (Cu, 1004 ± 5 μg mL^−1^), zinc (Zn, 1002 ± 3 μg mL^−1^), palladium (Pd, 1000 ± 3 μg mL^−1^), aluminum (Al, 997 ± 4 μg mL^−1^), tin (Sn, 1003 ± 4 μg mL^−1^), lead (Pb, 1001 ± 4 μg mL^−1^), and yttrium (Y, 1005 ± 5 μg mL^−1^) were purchased from SCP SCIENCE. A 67–70% w/w HNO_3_ solution (trace metal analysis) was purchased from VWR. Ultrapure water (18.2 MΩ cm) from a Purelab Flex system was used for preparing solutions.

### DES and stock solution preparation

Three different DES matrices were investigated: ChCl:EG:I_2_, ChCl:EG, and ChCl:LA. To prepare the DES, ChCl was mixed with EG or LA at a 1 : 2 molar ratio. ChCl was dried under vacuum for 24 h before being used, while EG, LA, and I_2_ were used as received. The mixture was stirred at 80 °C until the formation of a homogeneous liquid, namely the DES (Fig. 1B). The resulting DES was diluted 10 times with 5% w/w HNO_3_ and was used as the blank as well as for dilutions in all subsequent steps of this work. The 5% w/w HNO_3_ was prepared by dilution of the 67–70% w/w HNO_3_ solution with ultrapure water.

A multi-element 100 μg mL^−1^ calibration stock solution was prepared using the single element calibration solutions and the HNO_3_ diluted DES. An internal standard was used to overcome factors that could affect the behavior of our samples, such as viscosity, acid content, and fine particles.^31–33^ A 2 μg mL^−1^ yttrium internal standard was prepared by dilution of the yttrium single element calibration solution in 5% w/w HNO_3_. A Hamilton Microlab 600 diluter/dispenser system was used to prepare all the standard solutions. The standard solutions were kept in 15 and 50 mL Falcon tubes and were prepared weekly.

The calibration standard solutions were prepared at nine concentration levels for every metal tested in this study: 0.01, 0.04, 0.1, 0.4, 1, 4, 10, 20, and 40 μg mL^−1^. These calibration standard solutions were prepared by diluting the 100 μg mL^−1^ calibration stock solution with the HNO_3_ diluted DES. Further, a 2 μg mL^−1^ solution was prepared from another 100 μg mL^−1^ stock solution and was used as a control solution.

### MP-AES analysis

The standard solutions and the internal standard were filtered with syringe filters (45 μm) before injected *via* two 0.38 mm tubes to an Agilent Technologies 4100^+^ MP-AES (4100 upgraded to a 4200 torch system) (Fig. 1C). The pump speed, uptake time, stabilization time, and rinsing time with 5% w/w HNO_3_ were set to 15 rpm, 15 s on the fast pump mode, 45 s, and 60 s, respectively. The nebulizer pressure was optimized for all metals before each analysis and each sample was replicated four times. The wavelength calibration was performed daily. Table 1 shows the selected wavelengths for each metal studied.

**Table 1:** Elemental wavelength used for MP-AES analysis

Metal	Wavelength (nm)	Type
Li	610.365	Analyte
Mg	285.213	Analyte
Fe	371.993	Analyte
Co	340.512	Analyte
Ni	352.454	Analyte
Cu	324.754	Analyte
Zn	213.857	Analyte
Pd	340.458	Analyte
Al	394.401	Analyte
Sn	317.505	Analyte
Pb	405.781	Analyte
Y	371.029, 324.227	Internal standard

## Method validation

Several international conferences and organizations have issued guidelines and protocols regarding single laboratory validation of an analytical method.^34^ In our study, the detection and quantification of metals dissolved in DES were evaluated considering the International Council for Harmonization (ICH) guidelines,^35^ AOAC International (AOAC),^36^ the International Union of Pure and Applied Chemistry (IUPAC),^42^ the protocol for the validation of an analytical method in chemistry by the Centre of expertise in environmental analysis of Québec,^37^ as well peer-reviewed literature.^34,37–39^ The validation parameters considered in our work included linearity, limits of detection and quantification, accuracy, precision, and selectivity (Fig. 1C). Microsoft Excel and Sigma Plot software were used.

### Linearity

A calibration curve was prepared by plotting intensity *versus* concentration data for the studied metal concentrations and performing a linear regression, *ŷ* = *ax* + *b*, where *ŷ* is the predicted value, *a*, is the slope, and *b* is the *y*-intercept.^34,35,37^ Linearity can be defined with at least four concentration levels, but most guidelines consider six as acceptable.^34,35^ In this study, nine concentration levels were used to assess linearity and both matrix-containing solutions and blanks were used.^34,35,40^ The linearity of the calibration curves was first assessed *via* the calculation of the correlation coefficients (*R*^2^), which offers limited insights according to literature.^33,41,42^ To confirm linearity, the relative residuals were calculated^31,33,34,39,40,42^ and the relative residual deviation needed to be less than 20%.^33,41^ To further confirm the linearity of the calibration curves, lack-of-fit tests (*F*-test) were carried out.^39,43^

### Limits of detection (LOD) and quantification (LOQ)

There are several methods available to estimate the LOD and LOQ, which correspond to the lowest concentration of analyte that can be consistently detected and the lowest concentration of analyte that can be detected consistently and accurately, respectively.^33,34,38,44^ In this work, LOD and LOQ values for each metal were estimated mathematically from the slope of linear calibration curve (slope_*m*_) and the standard deviation of the response (*i.e.*, positive intensity) of ten blank solutions (*σ*_blank_) (Eqn (1) and (2)).^6,30,45,46^1
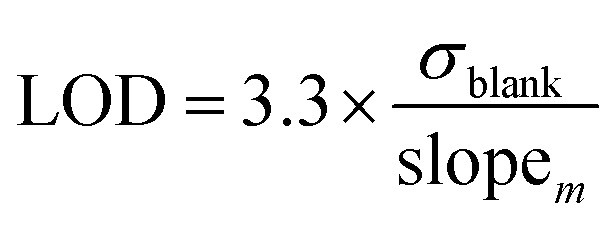
2
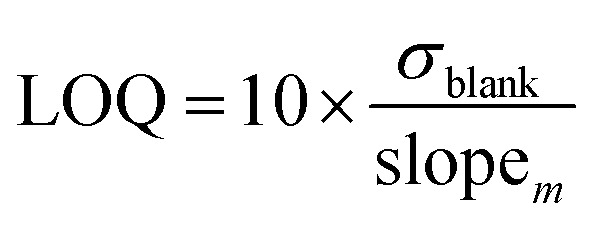


### Accuracy and precision

Systematic and random errors were estimated by assessing the method's accuracy and precision, respectively. The accuracy of the method was calculated as % recovery, namely the ratio of the measured concentration obtained from the linear calibration curve over the theoretical concentration value for each metal at three concentration levels: 2, 3, 5 μg mL^−1^. Accuracy values between 90% to 110% were considered acceptable.^32,33,38^ The method's random error was estimated by considering both intra- and inter-run precision. Intra-run precision, namely the repeatability of the method, was expressed as the relative standard deviation (RSD) and was the standard deviation over the mean value for four runs of the 2, 3, and 5 μg mL^−1^ samples for each metal. Inter-run precision, namely the intermediate precision of the method, which refers to the precision achieved within a single laboratory over a long period of time^38,39,44^ was reported as the pooled RSD (RSD_pooled_) of measurements obtained over a two-month period.

### Selectivity

In multi-elemental analyses with complicated matrices as in this study, samples may contain compounds which may interfere with the desired elemental quantification by analytical instruments. Selectivity ensures that these interferences, which may result from changes in the matrix, do not significantly affect the method's accuracy and precision.^39^ In this work, the linearity, LOD, LOQ, accuracy, and precision of the proposed method to measure Li, Co, Ni, Cu, and Al, were investigated in three different DES matrices: ChCl:EG:I_2_, ChCl:EG, and ChCl:LA. Finally, the accuracy and precision for 2 μg mL^−1^ samples of Li, Co, Ni, Cu, and Al dissolved in the three DES were measured and compared to that of 2 μg mL^−1^ aqueous solution samples, based on a calibration generated using aqueous standard solutions dissolved in HNO_3_ without the presence of DES. This was an important assessment of the importance of our method, as it highlighted the potential errors during the analysis of metals dissolved in DES without the use of the proposed method.

## Results and discussion

### Linearity

**Fig. 2:**
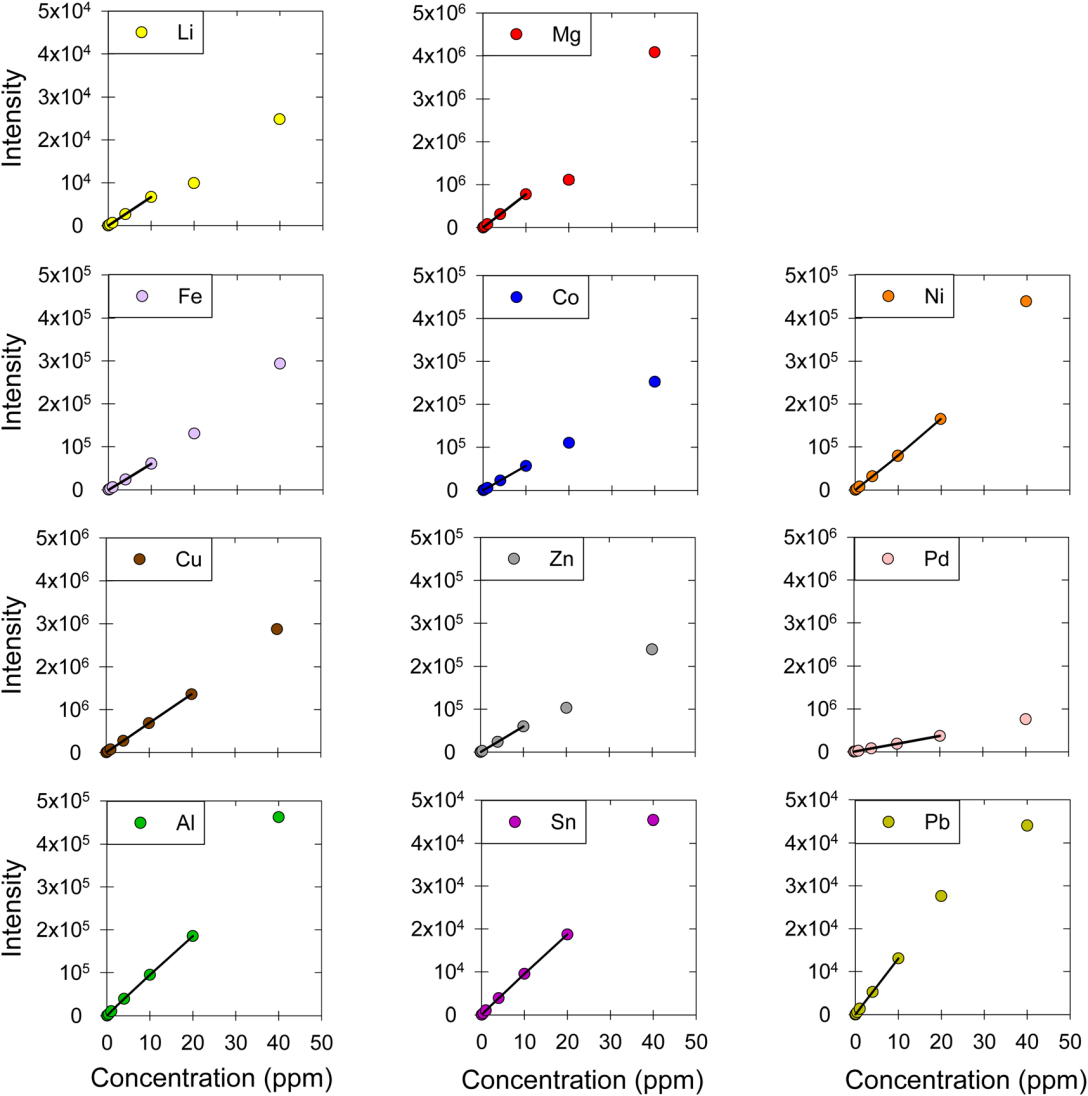
Calibration curves for Li, Mg, Fe, Co, Ni, Cu, Zn, Pd, Al, Sn, and Pb in ChCl:EG:I_2_. The markers represent the data points corresponding to the intensity-concentration values of each sample. The black straight line represents the linear range of the calibration curve. ESI, Fig. S1† shows the graphs between 0 and 1 ppm, which include the results from four concentration levels (0.01, 0.04, 0.1, 0.4, 1 μg mL^−1^).

The concentration of a series of metals dissolved in DES was measured (Fig. 2). Specifically, we quantified the concentration of alkali metals: lithium (Li); alkaline earth metals: magnesium (Mg); transition metals: iron (Fe), cobalt (Co), nickel (Ni), copper (Cu), zinc (Zn), palladium (Pd); and post-transition metals: aluminum (Al), tin (Sn), and lead (Pb). The linearity of the calibration curves was examined; the calibration curves were obtained using standard solutions at nine concentration levels: 0.01, 0.04, 0.1, 0.4, 1.0, 4.0, 10, 20, and 40 μg mL^−1^ (Fig. 2). Then, the linear ranges for each metal were determined based on the correlation coefficients (*R*^2^), residual plots, and *F*-tests. For all the metals, *R*^2^ was higher than 0.999, which is a good but not universally acceptable indicator of linearity.^30,33,38,39,47^ The relative residual was consistently lower than 10% in our work, which is acceptable based on literature.^10,33^ Further, to confirm the linearity of our calibration curves, an F-test was carried out;^39,41,48,49^ a comparison between *F*_calculated_ and *F*_tabulated_ values led to the elimination of calibration points that fall outside the linear range and the determination of the linear range for each metal (Fig. 2). The linear regression equations, *R*^2^ values, the *F*-test results, as well as the linear ranges are presented in detail in Table 2.

**Table 2:** Linear regression equations, correlation coefficients (*R*^2^), *F*-test values, linear ranges, and LOD and LOQ for the calibration curves of Li, Mg, Fe, Co, Ni, Cu, Zn, Pd, Al, Sn, and Pb in ChCl:EG:I_2_

Metal	Linear regression equation	*R* ^2^	*F*_calculated_ < *F*_tabulated_	Linear range (ppm)	LOD (ppm	LOQ (ppm)
Li	*y* = 669.77*x* + 2.91	1.0000	0.10 < 2.93	0.04–10.00	0.006	0.020
Mg	*y* = 77 676.00*x* + 173.97	1.0000	3.09 < 3.29	0.10–10.00	0.071	0.220
Fe	*y* = 6073.00*x* − 2.26	1.0000	0.12 < 3.71	0.10–10.00	0.030	0.091
Co	*y* = 5701.30*x* + 45.62	1.0000	2.73 < 2.93	0.04–10.00	0.004	0.011
Ni	*y* = 8260.50*x* − 474.56	0.9997	3.01 < 3.26	0.10–20.00	0.040	0.110
Cu	*y* = 68 317.00*x* + 1818.71	1.0000	0.06 < 2.93	0.10–20.00	0.003	0.008
Zn	*y* = 5970.30*x* − 79.85	1.0000	0.36 < 4.46	0.10–10.00	0.040	0.115
Pd	*y* = 18 305.00*x* + 1894.51	0.9999	1.10 < 2.93	0.10–20.00	0.003	0.009
Al	*y* = 9262.10*x* + 741.50	0.9998	1.76 < 2.93	0.10–20.00	0.007	0.020
Sn	*y* = 933.91*x* + 74.99	0.9999	0.60 < 3.29	0.40–20.00	0.011	0.033
Pb	*y* = 1302.00*x* + 20.70	1.0000	0.14 < 3.26	0.04–10.00	0.020	0.060

The linear range was found to be up to 10 ppm for Li, Mg, Fe, Co, Zn, and Pb and up to 20 ppm for Ni, Cu, Pd, Al, and Sn. Generally, a good linear range is considered to include at least four concentration levels.^10^ The linear range reported in this work for all metals was good, as it was always over five concentration levels. Despite the difference between the matrices, this method's linear ranges are comparable to other methods that investigated dissolved metals in aqueous matrices using MP-AES, as the reported linear ranges for Cu, Fe, Mg, Zn are up to 20 ppm.^21,50,51^

### LOD and LOQ

The LOD and LOQ values for each metal are presented in Table 2. MP-AES has high sensitivity as an analytical instrument, which can explain the lower or comparable LOD of the proposed method compared to similar methods using F-AAS and ICP-AES.^10,13,44^ The LOD and LOQ values were estimated four times in four months. The highest values are reported in Table 2 as a conservative approach to assure the concentration values are reliable. The LOD and LOQ for our method are consistently comparable to other studies on metals detection using MP-AES.^21,52–55^

### Accuracy and precision

**Fig. 3:**
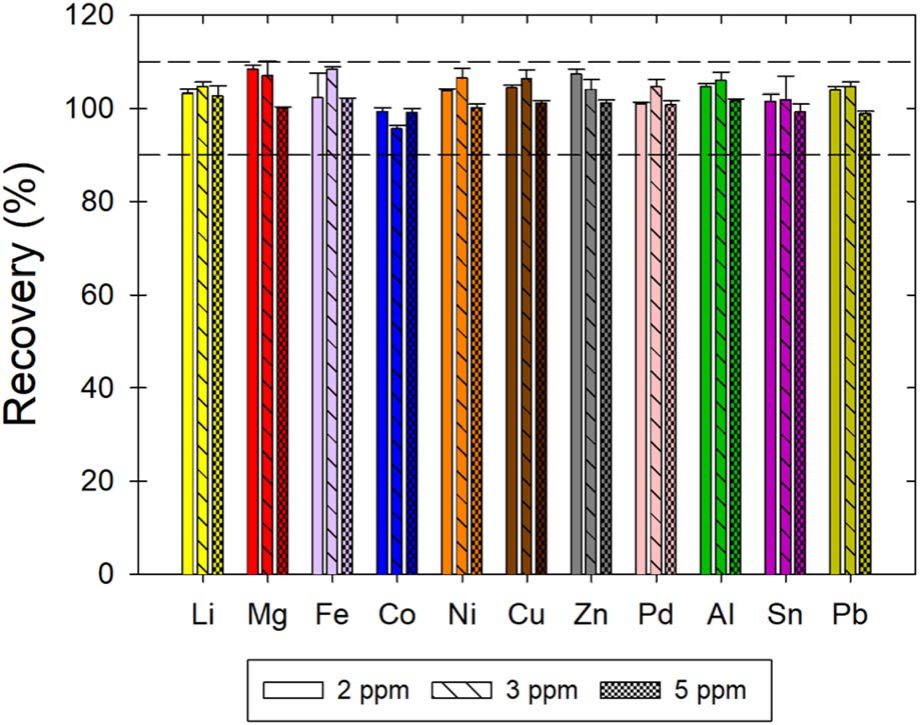
Recovery of Li, Mg, Fe, Co, Ni, Cu, Zn, Pd, Al, Sn, and Pb in ChCl:EG:I_2_. The acceptability criterion for the recovery was between 90 and 110% as indicated by the horizontal dotted lines.

Accuracy was estimated by calculating recovery of a metal at three concentration levels, *i.e.*, 2, 3, and 5 μg mL^−1^. The recovery was in the acceptable range of 90% to 110% for all metals (Fig. 3). The average recoveries for each metal as well as the average repeatability and intermediate precision results are summarized in Table 3.

**Table 3:** Accuracy, repeatability (RSD), and intermediate precision (RSD_pooled_) of Li, Mg, Fe, Co, Ni, Cu, Zn, Pd, Al, Sn, and Pb dissolved in ChCl:EG: I_2_. Accuracy is reported as the mean value of four replicates and RSD was calculated from four replicates. The RSD_pooled_ was calculated from four daily replicate samples acquired on 17 different days

Metal	Concentration (ppm)	Accuracy recovery (%) ± SD (%)	Repeatability RSD (%)	Intermediate precision RSD_pooled_ (%)
Li	2	103.30 ± 0.79	0.78	1.27
	3	104.66 ± 0.95	0.91	—
	5	102.70 ± 2.18	2.12	—
Mg	2	108.35 ± 0.91	0.84	2.51
	3	107.02 ± 3.19	2.99	—
	5	100.19 ± 0.26	0.26	—
Fe	2	102.33 ± 5.18	5.06	0.72
	3	108.40 ± 0.49	0.49	—
	5	102.20 ± 0.09	0.09	—
Co	2	99.37 ± 0.85	0.86	3.67
	3	95.67 ± 0.74	0.77	—
	5	99.17 ± 0.88	0.89	—
Ni	2	103.81 ± 0.40	0.39	1.83
	3	106.42 ± 2.15	2.02	—
	5	100.18 ± 0.79	0.78	—
Cu	2	104.04 ± 0.62	0.59	1.28^aa^
	3	106.30 ± 1.93	1.81	—
	5	101.20 ± 0.49	0.49	—
Zn	2	107.31 ± 1.105	1.03	2.55
	3	104.03 ± 2.12	2.12	—
	5	101.23 ± 0.59	0.59	—
Pd	2	101.01 ± 0.33	0.33	1.33
	3	104.61 ± 1.63	1.56	—
	5	100.84 ± 0.93	0.93	—
Al	2	104.69 ± 0.56	0.53	1.31
	3	105.97 ± 1.83	1.73	—
	5	101.66 ± 0.46	0.45	—
Sn	2	101.60 ± 1.46	1.44	2.38
	3	101.92 ± 5.01	4.91	—
	5	99.36 ± 1.63	1.64	—
Pb	2	103.88 ± 0.75	0.72	1.20
	3	104.65 ± 01.01	0.97	—
	5	99.03 ± 0.41	0.42	—

a Calculated from four daily replicates samples measured on 40 different days.

**Fig. 4:**
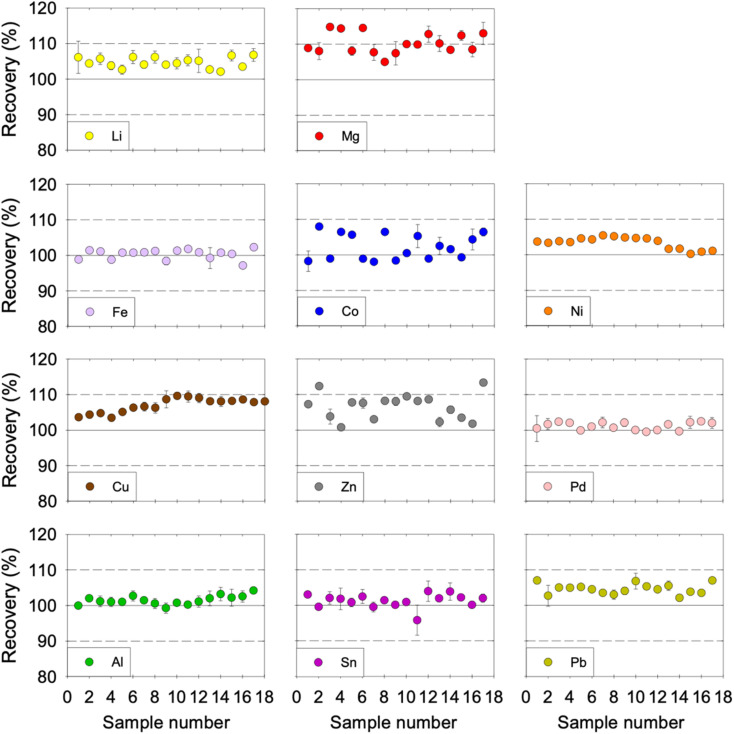
Inter-run precision of Li, Mg, Fe, Co, Ni, Cu, Zn, Pd, Al, Sn, and Pb in ChCl:EG:I_2_. Over a two-month period, 17 samples with 2 μg mL^−1^ concentration were prepared on different days and measured. The acceptability criterion for the recovery was between 90 and 110% as indicated by the horizontal dotted lines. Cu was tested for an extended period of time, namely 40 samples prepared on different days, and the results are presented in ESI, Fig. S2.†

The repeatability (RSD) and the intermediate precision (RSD_pooled_) were evaluated to determine the precision of the developed method. The RSD was estimated by measuring four replicate samples for each concentration level in the same day. The RSD for the tested concentration levels and metals was acceptable as it was less than 5.1%. The RSD_pooled_ of each metal was evaluated over a two-month period, whereby 17 samples were obtained and analysed from different days. The RSD_pooled_ for each metal was below 3.7%, which indicates that our method has an acceptable intermediate precision (Table 3). The recovery of each metal over the two-month period was acceptable, as shown in Fig. 4. That said, Mg, Co, and Zn showed noticeable variability in the inter-run precision results. Despite that fact, the recovery of Co and Zn was consistently within the acceptable range (with only two exceptions for Zn that had 112% and 113% recovery). However, the recovery of Mg was consistently close to the higher 110% acceptability limit and surpassed it in six out of the 17 samples tested but remained consistently below 115%. Therefore, the proposed method could be described as accurate and precise for the analysis of the eleven dissolved metals in ChCl:EG:I_2_ DES.

### Selectivity

**Fig. 5:**
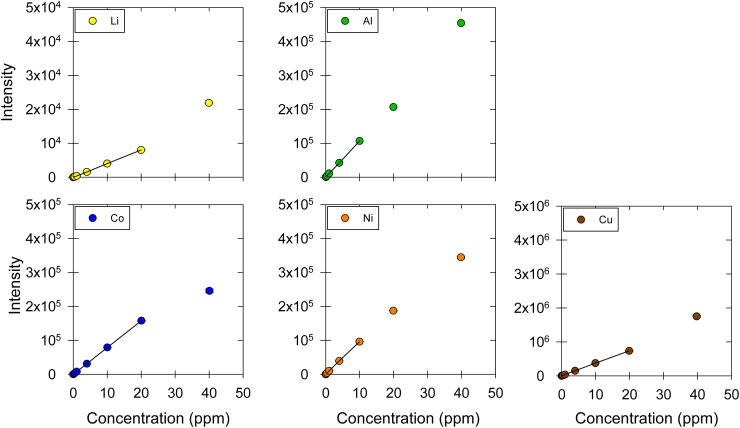
Calibration curves for Li, Co, Ni, Cu, and Al in ChCl:EG. The markers represent the data points corresponding to the intensity–concentration values of each sample. The black straight line represents the linear range of the calibration curve. ESI, Fig. S3† shows the results at the 0.01, 0.04, 0.1, 0.4, 1 μg mL^−1^ concentration levels, which are contracted in this figure.

**Fig. 6:**
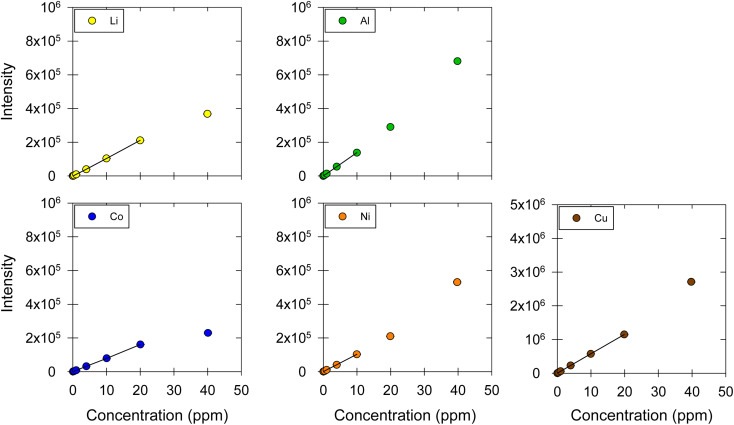
Calibration curves for Li, Co, Ni, Cu, and Al in ChCl:LA. The markers represent the data points corresponding to the intensity–concentration values of each sample. The black straight line represents the linear range of the calibration curve. ESI, Fig. S4† shows the results at the 0.01, 0.04, 0.1, 0.4, 1 μg mL^−1^ concentration levels, which are contracted in this figure.

**Fig. 7:**
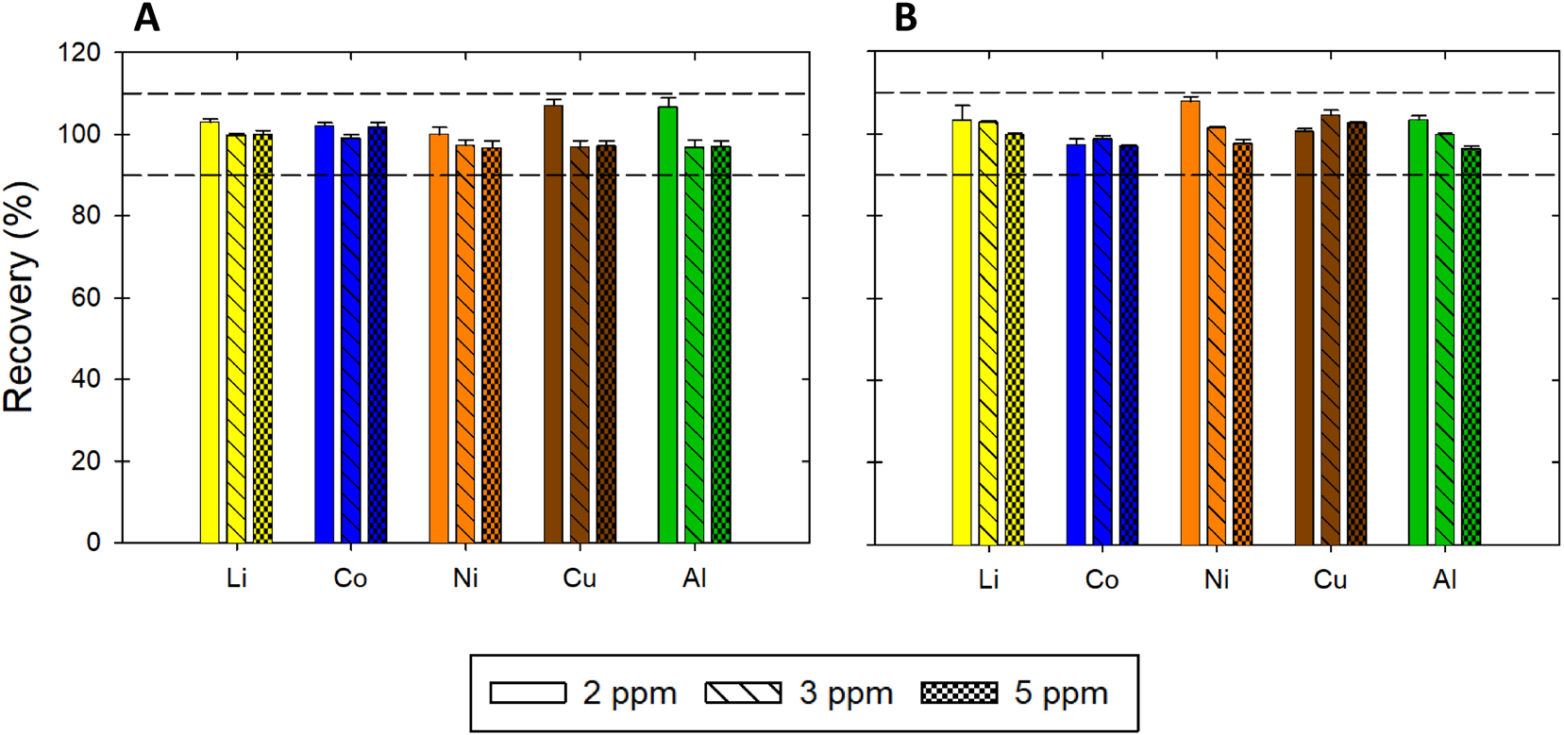
Recovery of Li, Co, Ni, Cu, and Al in (A) ChCl:EG and (B) ChCl:LA. The acceptability criterion for the recovery was between 90 and 110%, as indicated by the horizontal dotted lines.

The selectivity was evaluated by testing two different DES matrices: (1) ChCl : EG with a 1 : 2 molar ratio without I_2_ and (2) ChCl : LA with a 1 : 2 molar ratio. Five metals were used to assess the matrix effect: Li, Co, Ni, Cu, and Al. For each matrix, the calibration curves were determined; the linear ranges were proposed based on *R*^2^, relative residuals, and *F*-tests; LOD and LOQ were estimated; and the recovery and repeatability were calculated for three concentration levels (*i.e.*, 2, 3, and 5 μg mL^−1^). The calibration curves obtained for the ChCl : EG and ChCl:LA DES matrices are shown in Fig. 5 and 6, respectively. Table 4 lists the linear regression equations, *R*^2^, *F*-test values, linear ranges, and LOD and LOQ results. The recovery and repeatability of the five metals in the ChCl:EG and ChCl:LA DES matrices were acceptable, as indicated by the results presented in Table 5 and Fig. 7.

**Table 4:** Linear regression equations, correlation coefficients (*R*^2^), *F*-test values, and linear ranges for the calibration curves of Li, Co, Ni, Cu, and Al in ChCl:EG and ChCl:LA

Metal	DES	Linear regression equation	*R* ^2^	*F*_calculated_ < *F*_tabulated_	Linear range (ppm)	LOD (ppm)	LOQ (ppm)
Li	ChCl:EG	*y* = 403.27*x* − 18.57	1.0000	0.70 < 2.68	0.04–20.00	0.022	0.070
	ChCl:LA	*y* = 10 631.10*x* − 1530.36	0.9999	1.69 < 2.93	0.10–20.00	0.001	0.002
Co	ChCl:EG	*y* = 7893.80*x* + 143.38	1.0000	0.23 < 2.68	0.04–20.00	0.004	0.013
	ChCl:LA	*y* = 78 029.86*x* − 237.43	0.9999	0.79 < 3.29	0.40–20.00	0.009	0.031
Ni	ChCl:EG	*y* = 9632.31*x* + 522.85	0.9999	3.02 < 3.26	0.10–10.00	0.014	0.042
	ChCl:LA	*y* = 10 359.73*x* − 80.69	1.0000	0.58 < 3.29	0.10–10.00	0.003	0.011
Cu	ChCl:EG	*y* = 37 150.00*x* + 1425.40	0.9999	0.87 < 2.93	0.10–20.00	0.001	0.030
	ChCl:LA	*y* = 57 834.81*x* − 310.88	1.0000	0.04 < 2.93	0.10–20.00	0.003	0.008
Al	ChCl:EG	*y* = 10 699.00*x* + 101.81	1.0000	0.79 < 3.29	0.04–9.96	0.038	0.115
	ChCl:LA	*y* = 13 850.00*x* + 84.872	1.0000	0.08 < 2.68	0.00–9.96	0.005	0.017

**Table 5:** Accuracy and repeatability of Li, Co, Ni, Cu, and Al in ChCl:EG and ChCl:LA

Metal	Concentration (ppm)	Accuracy recovery (%) ± SD (%)		Repeatability RSD (%)	
		ChCl:EG	ChCl:LA	ChCl:EG	ChCl:LA
Li	2	103.00 ± 0.71	103.37 ± 3.54	0.69	3.43
	3	99.75 ± 0.50	102.83 ± 0.43	0.50	0.42
	5	100.00 ± 0.99	99.85 ± 0.34	0.99	0.34
Co	2	102.12 ± 0.75	97.37 ± 1.38	0.73	1.41
	3	99.08 ± 0.96	98.83 ± 0.64	0.97	0.65
	5	101.90 ± 1.04	96.95 ± 0.38	1.02	0.39
Ni	2	100.02 ± 1.70	107.89 ± 1.23	1.70	1.18
	3	97.32 ± 1.37	101.44 ± 0.32	1.41	0.32
	5	96.63 ± 1.70	97.64 ± 0.90	1.76	0.92
Cu	2	107.11 ± 1.44	100.59 ± 0.66	1.35	0.66
	3	96.92 ± 1.55	104.59 ± 1.23	1.59	1.18
	5	97.19 ± 1.20	102.68 ± 0.15	1.23	0.15
Al	2	106.07 ± 2.29	103.40 ± 1.05	2.16	1.02
	3	96.77 ± 1.86	99.85 ± 0.36	1.92	0.36
	5	97.01 ± 1.44	96.34 ± 0.73	1.48	0.76

The sensitivity for Li, Co, Ni, and Al was higher in the ChCl:LA compared to the ChCl:EG and ChCl:EG:I_2_. Only for Cu the highest sensitivity was measured in the ChCl:EG:I_2_. The sensitivity increased for Li and Cu in the presence of I_2_ but decreased for Co, Ni and Al. Also, the upper limit of the linear ranges for Li and Co in ChCl:EG and ChCl:LA were double compared to the ranges obtained with ChCl:EG:I_2_. However, the upper limit was half for Ni and Al in ChCl:EG and ChCl:LA compared to ChCl:EG:I_2_. The linear range for Cu remained the same for all three DES. The linear range in ChCl:EG and ChCl:LA for all five metals was the same. The LOD and LOQ were also affected by the change in the matrix, but no specific trend was observed. Overall, the proposed method is accurate and precise for different matrices, but changing the matrix is expected to change the linear range, sensitivity, LOD and LOQ.

### Importance of DES matrix-matched calibration

**Fig. 8:**
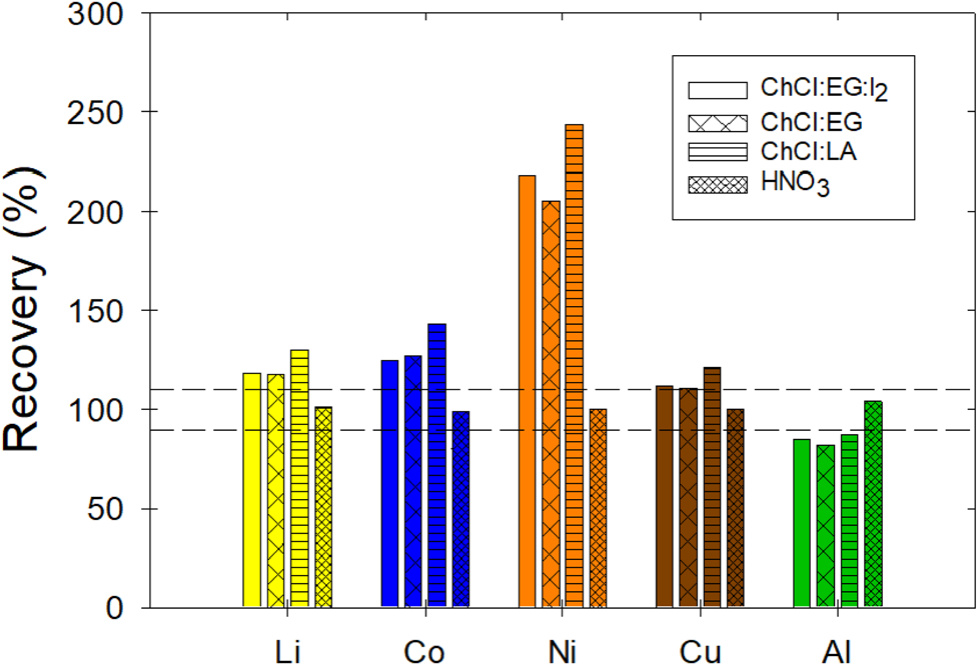
Recovery of 2 μg mL^−1^ samples of Li, Co, Ni, Cu, and Al in ChCl:EG:I_2_, ChCl:EG, ChCl:LA, and HNO_3_ when the calibration was done using non-matrix-matched standards, *i.e.*, aqueous standard solutions based on HNO_3_. The acceptability criterion for the recovery was between 90 and 110%, as indicated by the horizontal dotted lines.

To assess the significance of the proposed method in terms of potential errors caused by non-matrix-matched calibrations, the recovery and repeatability (RSD) of 2 μg mL^−1^ samples of Li, Co, Ni, Cu, and Al dissolved in three DES was measured based on a calibration done using aqueous HNO_3_ standard solutions without the presence of DES. The recovery was unacceptable as it failed to meet the acceptability criteria for all metals in all three DES samples (Fig. 8). In fact, the reported recovery values were as low as 82.04 ± 0.01% and as high as 243.65 ± 0.1%, thus indicating the importance of the developed method (Table 6). Yet, the repeatability was acceptable for all the metals tested and in all the DES matrices. To ensure that the aqueous standards and the MP-AES performed analyses were accurate, samples with 2 μg mL^−1^ of Li, Co, Ni, Cu, and Al in the HNO_3_ were measured; both the recovery and repeatability results were acceptable (Table 6 and Fig. 8). Therefore, the matrix can affect the accuracy of the method if matrix-matched calibration curves are not used. This highlights the important step in the proposed method to use matrix-matched calibration curves to achieve accurate and precise measurements of metals in choline chloride based DES. In fact, interferences due to matrix effects have been reported in AAS, ICP-AES, and MP-AES methods; the use of matrix-matched calibrations has been suggested to minimize matrix effects.^44^ Therefore, the proposed method is expected to ensure the generation of reliable data that will allow the further development of solvometallurgical processes.

**Table 6:** Accuracy and repeatability Li, Co, Ni, Cu, and Al in ChCl:EG:I_2_, ChCl:EG, ChCl:LA and HNO_3_. The calibration was done using aqueous standard solutions in HNO_3_. The concentration of the samples was of 2 μg mL^−1^. In bold are values that fail the acceptability criteria, which are recovery between 90 and 110% and RSD below 10%

	Metal	ChCl:EG + I_2_	ChCl:EG	ChCl:LA	HNO_3_
Recovery (%) ± SD (%), RSD (%)	Li	**118.40 ± 0.01**, 0.01	**117.90 ± 0.2**, 0.01	**130.15 ± 0.05**, 0.40	101.15 ± 0.02, 0.01
Recovery (%) ± SD (%), RSD (%)	Co	**125.20 ± 0.01**, 0.01	**127.60 ± 0.00**, 0.00	**143.05 ± 0.2,** 0.10	99.35 ± 0.01, 0.01
Recovery (%) ± SD (%), RSD (%)	Ni	**218.40 ± 0.01**, 0.00	**205.70 ± 0.02**, 0.10	**243.65 ± 0.1**, 0.00	100.25 ± 0.02, 0.02
Recovery (%) ± SD (%), RSD (%)	Cu	**112.00 ± 0.09**, 0.08	**111.00 ± 0.05**, 0.05	**121.15 ± 0.01**, 0.01	100.35 ± 0.01, 0.01
Recovery (%) ± SD (%), RSD (%)	Al	**85.00 ± 0.03**, 0.04	**82.04 ± 0.01**, 0.01	**87.50 ± 0.02**, 0.03	104.25 ± 0.03, 0.03

## Conclusions

In this study, we propose and validate an analytical method for the detection of eleven metals, *i.e.*, Li, Mg, Fe, Co, Ni, Cu, Zn, Pd, Al, Sn, and Pb, dissolved in choline chloride based deep eutectic solvents by microwave plasma atomic emission spectroscopy (MP-AES). The method was properly validated with respect to linearity, LOD, LOQ, accuracy, precision, and selectivity. Linearity was assessed by estimating correlation coefficients (*R*^2^) and relative residuals and performing *F*-tests, which helped to determine the linear ranges for each metal. The upper limit of the linear range for Li, Mg, Fe, Co, Zn, and Pb was 10 ppm, while for Ni, Cu, Pd, Al, and Sn reached 20 ppm. The metal with the lowest LOD and LOQ was Cu with reported values of 0.003 ppm and 0.008 ppm, respectively; Mg was the metal with the highest reported LOD and LOQ at 0.07 and 0.22 ppm, respectively. The repeatability and intermediate precision were acceptable and consistently less than 5.1%, while the recovery was acceptable as it was between 96–108% for all the metals studied. The selectivity was tested for two DES matrices, namely ChCl:EG and ChCl:LA and it was not found to affect the accuracy and precision of the proposed method. However, the linear ranges, sensitivity, and LOD and LOQ values changed when the matrix changed. To that extent, experiments were conducted to highlight the importance of using matrix-matched calibrations for accurate measurements, as suggested by the proposed method. Overall, the proposed method is accurate and precise for quantifying metals in choline chloride based DES. Therefore, it is expected that the proposed method will be widely used in solvometallurgy as we seek to produce metals *via* greener and more sustainable ways.

## Author contributions

Halimeh Askari Sabzkoohi: methodology, formal analysis, visualization, writing – original draft. Vicky Dodier: methodology, formal analysis, writing – review & editing. Georgios Kolliopoulos: writing – review & editing, visualization, resources, supervision, funding acquisition.

## Conflicts of interest

There are no conflicts to declare.

## Supplementary Material

RA-013-D3RA02372A-s001
