# Supplementary material for: A validated analytical method to measure metals dissolved in deep eutectic solvents
Source: RSC Adv. 2023 May 16;13(22):14887–98. doi: 10.1039/d3ra02372a (PMC10186337; doi:10.1039/d3ra02372a)
Supplement: RA-013-D3RA02372A-s001 [file RA-013-D3RA02372A-s001.pdf]

Electronic Supplementary Material (ESI) for RSC Advances.
This journal is © The Royal Society of Chemistry 2023

Supplementary Information

A validated analytical method to measure metals dissolved in deep eutectic solvents

Halimeh Askari Sabzkoohi, Vicky Dodier, Georgios Kolliopoulos*

Department of Mining, Metallurgical, and Materials Engineering,
Université Laval, 1065 Av. de la Médecine, Québec, Québec, Canada G1V 0A6

***Corresponding authors' email:** *georgios.kolliopoulos@gmn.ulaval.ca*

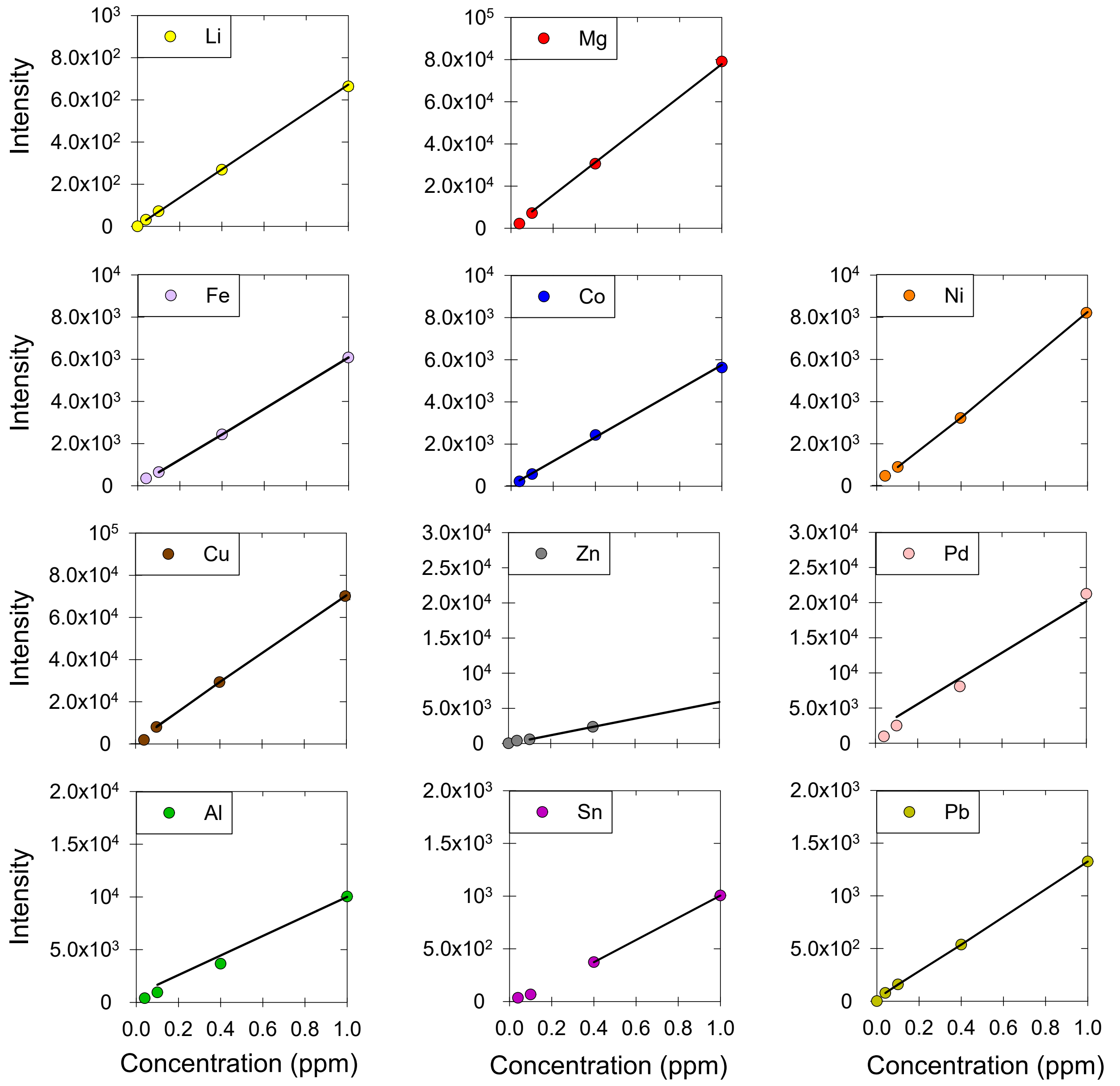

Figure S1: Calibration curve data at the 0.01, 0.04, 0.1, 0.4, 1 μg/mL concentration levels, which were contracted in Figure 2, for Li, Mg, Fe, Co, Ni, Cu, Zn, Pd, Al, Sn, and Pb in ChCl:EG:I_2. The markers represent the data points corresponding to the intensity-concentration values of each sample. The black straight line represents the linear part of each calibration curve, as obtained from the linear regression equations, R^2 values, and the F-test results.

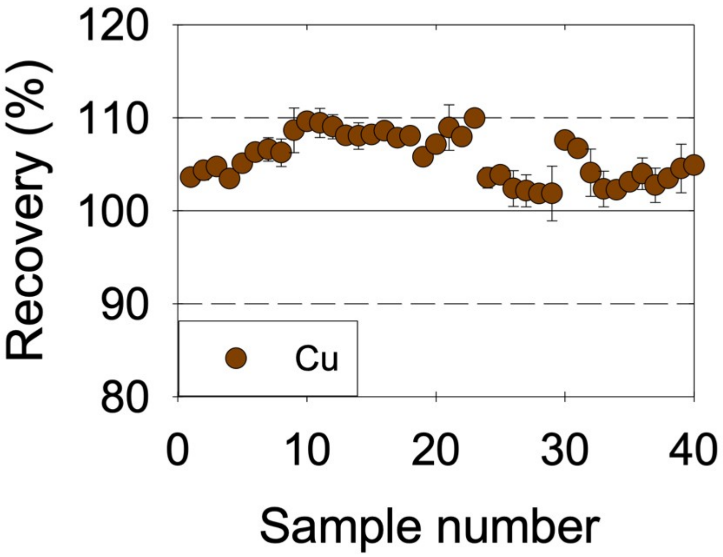

Figure S2: Inter-run precision of the analytical method developed for the determination of Cu in ChCl:EG:I_2. Measurements of 40 samples of 2 μg/mL were obtained at different days in a 2-month period and the accuracy (as % recovery) of the analytical method for each element was estimated. The acceptability criterion for the recovery was within 90–110%.

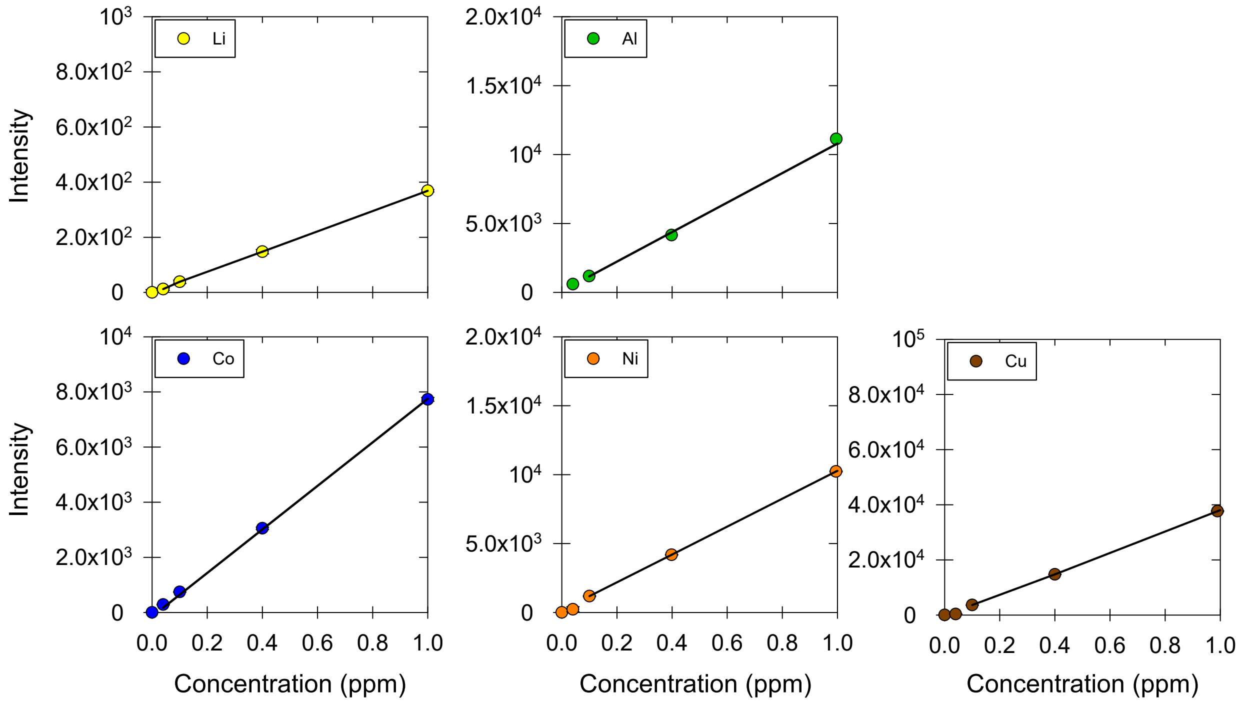

Figure S3: Calibration curve data at the 0.01, 0.04, 0.1, 0.4, 1 μg/mL concentration levels, which were contracted in Figure 5, for Li, Co, Ni, Cu, and Al in ChCl:EG. The markers represent the data points corresponding to the intensity-concentration values of each sample. The black straight line represents the linear part of each calibration curve, as obtained from the linear regression equations, R^2 values, and the F-test results.

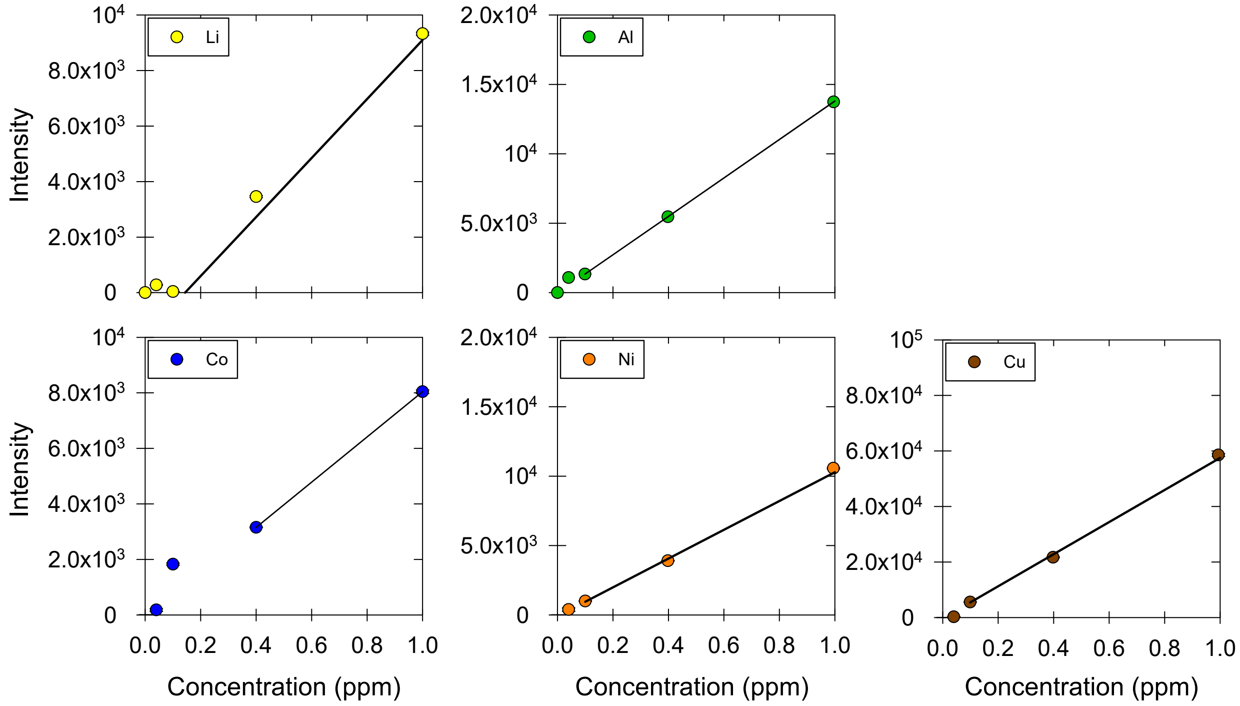

Figure S4: Calibration curve data at the 0.01, 0.04, 0.1, 0.4, 1 µg/mL concentration levels, which were contracted in Figure 6, for Li, Co, Ni, Cu, and Al in ChCl:LA. The markers represent the data points corresponding to the intensity-concentration values of each sample. The black straight line represents the linear part of each calibration curve, as obtained from the linear regression equations, R^2 values, and the F-test results.
